# Postural Muscle Unit Plasticity in Stroke Survivors: Altered Distribution of Gastrocnemius' Action Potentials

**DOI:** 10.3389/fneur.2019.00686

**Published:** 2019-06-26

**Authors:** Taian M. Vieira, Thiago Lemos, Laura A. S. Oliveira, Carlos H. R. Horsczaruk, Gabriel R. Freitas, Fernanda Tovar-Moll, Erika C. Rodrigues

**Affiliations:** ^1^Laboratorio di Ingegneria del Sistema Neuromuscolare (LISiN), Dipartimento di Elettronica e Telecomunicazioni, and PoliTo^BIO^Med Lab, Politecnico di Torino, Turin, Italy; ^2^Programa de Pós-Graduação em Ciências da Reabilitação, Centro Universitário Augusto Motta – UNISUAM, Rio de Janeiro, Brazil; ^3^Graduação em Fisioterapia, Instituto Federal de Educação, Ciência e Tecnologia – IFRJ, Rio de Janeiro, Brazil; ^4^Unidade de Conectividade Cerebral, Instituto D'Or de Pesquisa e Ensino – IDOR, Rio de Janeiro, Brazil; ^5^Programa de Pós-Graduação em Ciências Mofológicas, Instituto de Ciências Biomédicas, Universidade Federal do Rio de Janeiro – UFRJ, Rio de Janeiro, Brazil

**Keywords:** motor unit, electromyogram, stroke, gastrocnemius, standing

## Abstract

Neuromuscular adaptations are well-reported in stroke survivors. The death of motor neurons and the reinnervation of residual muscle fibers by surviving motor neurons, for example, seem to explain the increased density of muscle units after stroke. It is, however, unknown whether reinnervation takes place locally or extensively within the muscle. Here we combine intramuscular and surface electromyograms (EMGs) to address this issue for medial gastrocnemius (MG); a key postural muscle. While seven stroke survivors stood upright, two intramuscular and 15 surface EMGs were recorded from the paretic and non-paretic gastrocnemius. Surface EMGs were triggered with the firing instants of motor units identified through the decomposition of both intramuscular and surface EMGs. The standard deviation of Gaussian curves fitting the root mean square amplitude distribution of surface potentials was considered to assess differences in the spatial distribution of motor unit action potentials and, thus, in the distribution of muscle units between limbs. The median number of motor units identified per subject in the paretic and non-paretic sides was, respectively, 2 (range: 1–3) and 3 (1–4). Action potentials in the paretic gastrocnemius were represented at a 33% wider skin region when compared to the non-paretic muscle (Mann-Whitney; *P* = 0.014). Side differences in the representation of motor unit were not associated with differences in subcutaneous thickness (skipped-Spearman *r* = −0.53; confidence interval for *r*: −1.00 to 0.63). Current results suggest stroke may lead to the enlargement of the gastrocnemius muscle units recruited during standing. The enlargement of muscle units, as assessed from the skin surface, may constitute a new marker of neuromuscular plasticity following stroke.

## Introduction

Motor impairment is a widely recognized consequence of stroke (1). Structural changes in the spinal motor neuron and its muscle fibers have been advocated a contributing factor for the loss of motor control in stroke survivors (2, 3). Indeed, loss of motor neurons, muscle atrophy and fiber-type grouping have been reported within 2–5 months after stroke (2–5). It is the loss of motor neurons that seems to lead to major, structural changes within the paretic muscle. More specifically, muscle fibers belonging to dead motor neurons seem to be re-innervated by surviving ones. This structural change is substantiated by increases in the size of motor unit action potentials and, more directly, by collateral sprouting and greater fiber density at chronic, stroke stages (6–8). In virtue of the increased number of fibers per motor unit, it seems therefore reasonable to ask whether these fibers span a larger region within the paretic muscle.

Different methodologies have been designed to assess the location of individual muscle units; i.e., fibers of single motor units. Muscle units may be assessed directly by staining and then locating glycogen-depleted fibers. Although this procedure was applied successfully to study the topography of cat muscle units (9), applying it in human muscles is currently unviable. Tracking action potentials of single motor units with a needle electrode moving along a corridor transverse to the fibers' direction constitutes an alternative method for assessing muscle units in humans (10). For muscles with large, physiological cross-sectional areas, however, this technique would provide a limited view of muscle units, as different corridors would have to be scanned. Recently, we have shown the surface representation of action potentials of medial gastrocnemius (MG) motor units, identified with intramuscular electrodes during standing, reflects well the distribution of muscle units (11). Notwithstanding these different, existing means, none, however, seems to have been considered to quantify the localization of muscle units following stroke.

Here we, therefore, investigate whether stroke affects the structure of MG muscle units recruited during standing. We specifically ask: how diffusely does the amplitude of action potentials of single, MG motor units distribute on the skin of paretic and non-paretic limbs? Upright stance approach was chosen because postural instability is one of the leading motor impairment observed after stroke (12, 13). Moreover, we selected MG because it seems to be greatly affected by stroke (7, 14) and because of its functional relevance to balance control (11, 15, 16). If reorganization of muscle units takes place extensively along MG, then, we would expect to detect surface potentials along a larger skin region in the paretic limb.

## Methods

### Participants

Eight, ischemic stroke survivors were recruited (four females; range values; age: 47–64 years; height: 147–172 cm; body mass: 51–102 kg; months from stroke: 42–120). The presence of aphasia, cognitive impairment, other neurological diseases or lesions, rheumatologic or metabolic diseases, pregnancy, or any musculoskeletal disorders affecting the standing posture were exclusion criteria. All participants could stand upright without external support for 60 s. The experimental procedures were approved by the local Institutional Ethic Committee (reference number: 13611913.8.0000.5249). All participants gave written informed consent in accordance with the Declaration of Helsinki.

### Experimental Protocol

**Figure 1:**
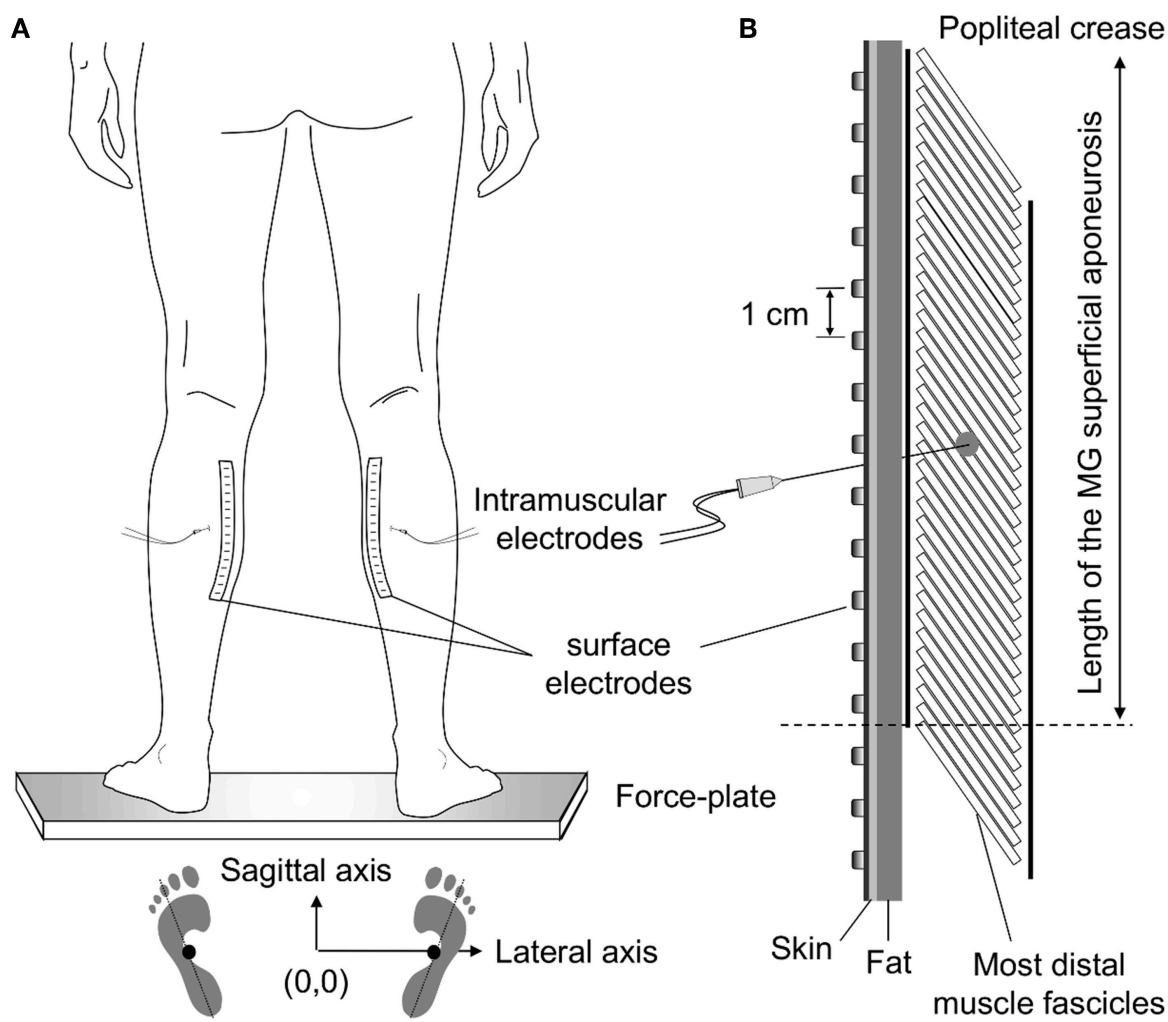
Standing posture and electrode positioning. While individuals stood at a comfortable stance **(A)** the feet position was marked on the force plate. These drawings were considered to quantify the approximate center of pressure (CoP) position corresponding to a symmetric weight distribution between limbs (see Methods, section Experimental Protocol). **(B)** shows the relative position between medial gastrocnemius (MG) and surface and intramuscular electrodes. Wire electrodes were inserted at the MG region corresponding to the central location between the most proximal, surface electrode, and the distal extremity of the superficial aponeurosis (see dashed line); for the example illustrated in panel **(B)**, this region roughly corresponds to the position of the sixth electrode from top to bottom. The length of MG superficial aponeurosis was estimated as the distance between its distal extremity and the popliteal crease.

**Figure 2:**
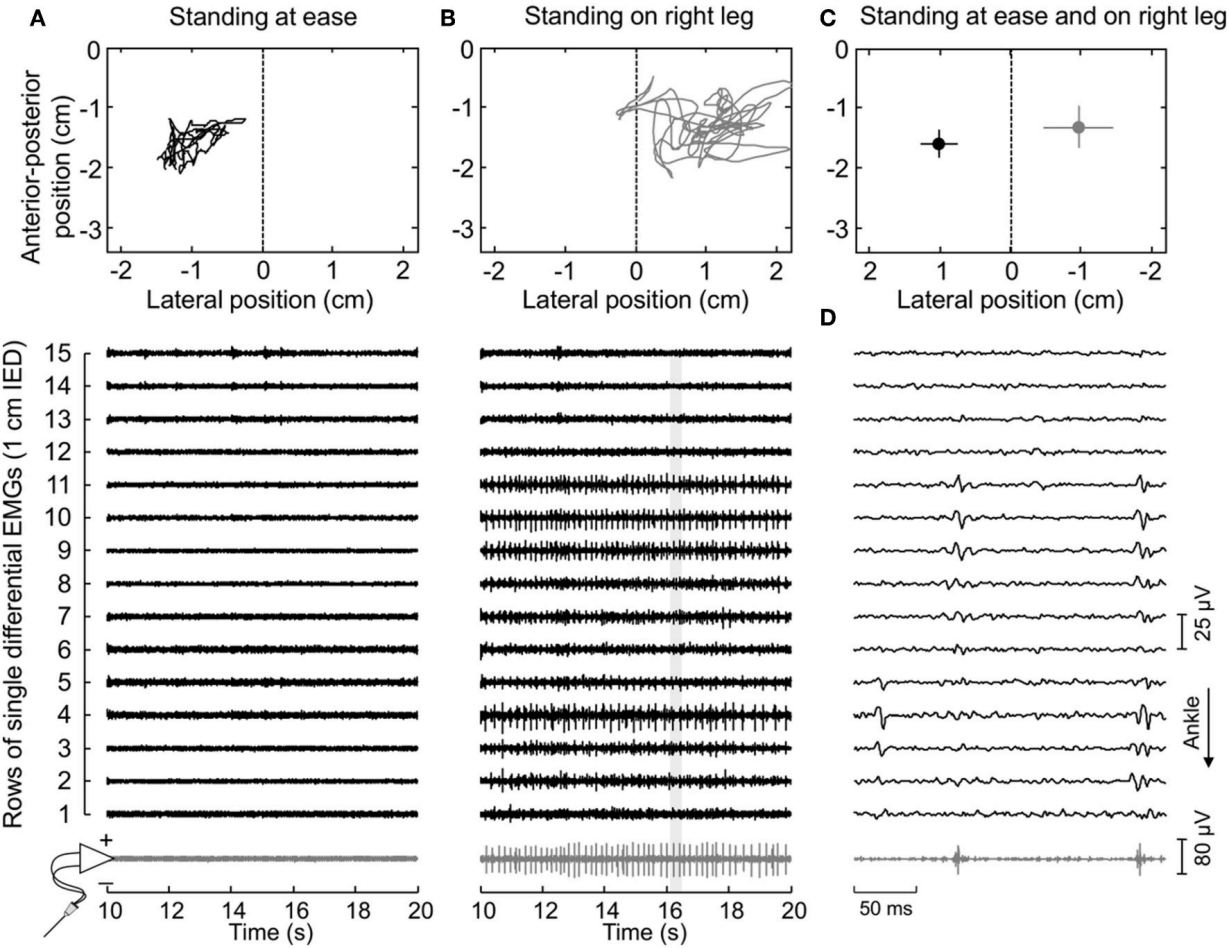
Muscle activity and standing posture. **(A)** illustrates the CoP position along the lateral and anterior-posterior axes (top panel) and the surface (black traces) and intramuscular (gray traces) electromyograms (EMGs) detected during 10 s for a representative subject. EMGs are shown exclusively for the paretic (right) MG. The dashed line in the top panel denotes the CoP location where body weight distributes roughly symmetrically between limbs (Figure 1A). Note that action potentials are not present both in the surface and intramuscular recordings. When this subject shifted his CoP toward the paretic limb **(B)**, action potentials could be clearly appreciated. An expanded view of EMGs (light gray rectangle) is shown in **(D)**. Note there is a correspondence in the instants when action potentials were detected by the intramuscular electrodes and by the central though not by the distal nor proximal electrodes in the surface array. **(C)** shows the mean CoP position, calculated over the entire recording (60 s), while the subject stood at ease (black circle) and on his right leg (gray circle). Horizontal and vertical traces correspond to the standard deviation along the lateral and sagittal directions, respectively.

Participants were asked to stand upright over a force-plate (AccuSwayPLUS, AMTI, Massachusetts, USA; Figure 1A), with their feet at a comfortable position. They were instructed to hang their arms loose alongside the body and to stand comfortably, without gross movements and without moving their feet. Two experimenters stood close to subjects at all times, to assist them in case of balance loss. Recordings started once subjects got acquainted with the standing tasks. At least two trials were applied, lasting 60 s each and with 5 min intervals. Given action potentials were occasionally not observed in both surface and intramuscular EMGs from the paretic limb in two subjects, additional trials were applied. Specifically, we provided these participants with visual feedback of their center of pressure (CoP) position and asked them to move it toward the paretic limb. After roughly 5 min of familiarization, participants could successfully load the paretic limb with at least 50% of their body weight (Figures 2A–C), according to the linear relationship between CoP lateral position and weight distribution between limbs (17). One trial per participant was retained for analysis; that providing the greatest number of clearly visible action potentials in surface EMGs detected from both sides. One participant did not show any motor unit action potential even when loading the paretic limb; signals recorded for this subject were disregarded.

### EMG Recordings

Intramuscular EMGs were detected with two pairs of Teflon-coated, stainless-steel wire electrodes (0.2 mm diameter; A-M Systems, Carlsborg, WA). Each pair was inserted in the paretic and non-paretic MG with a 25-gauge hypodermic needle. Two arrays of 16 silver bar electrodes each (1 × 10 mm; 10 mm inter-electrode distance; Spes-Medica, Battipaglia, Italy) were used to record 15 differential surface EMGs from each MG. Arrays were fixed to the skin with bi-adhesive pads filled with conductive paste (TEN 20 Conductive Paste, Weaver).

Wire and surface electrodes were positioned at specific MG locations (Figure 1). Initially, the location of the distal extremity of MG superficial aponeurosis [dashed line in Figure 1B (11)] was identified with ultrasound imaging (see “Muscle architecture” section). After that, the distance between this location and the popliteal crease was measured and marked on the skin. Wires were inserted halfway this distance. To ensure the tip of the wires would be located roughly beneath the surface array, needles were inserted obliquely to the skin. Finally, after shaving and cleaning the skin with abrasive paste, surface electrodes were aligned parallel to the muscle longitudinal axis. Amplification factor for both intramuscular and surface recordings ranged from 1,000 to 10,000 between participants (10–4,400 Hz bandwidth amplifier, EMG-USB2, OT-Bioelettronica, Turin, Italy). EMGs and ground reaction forces were digitized synchronously at 10,240 Hz (12 bits A/D converter; EMG-USB2, OT-Bioelettronica, Turin, Italy).

### Estimating the Surface Representation of Motor Units

**Figure 3:**
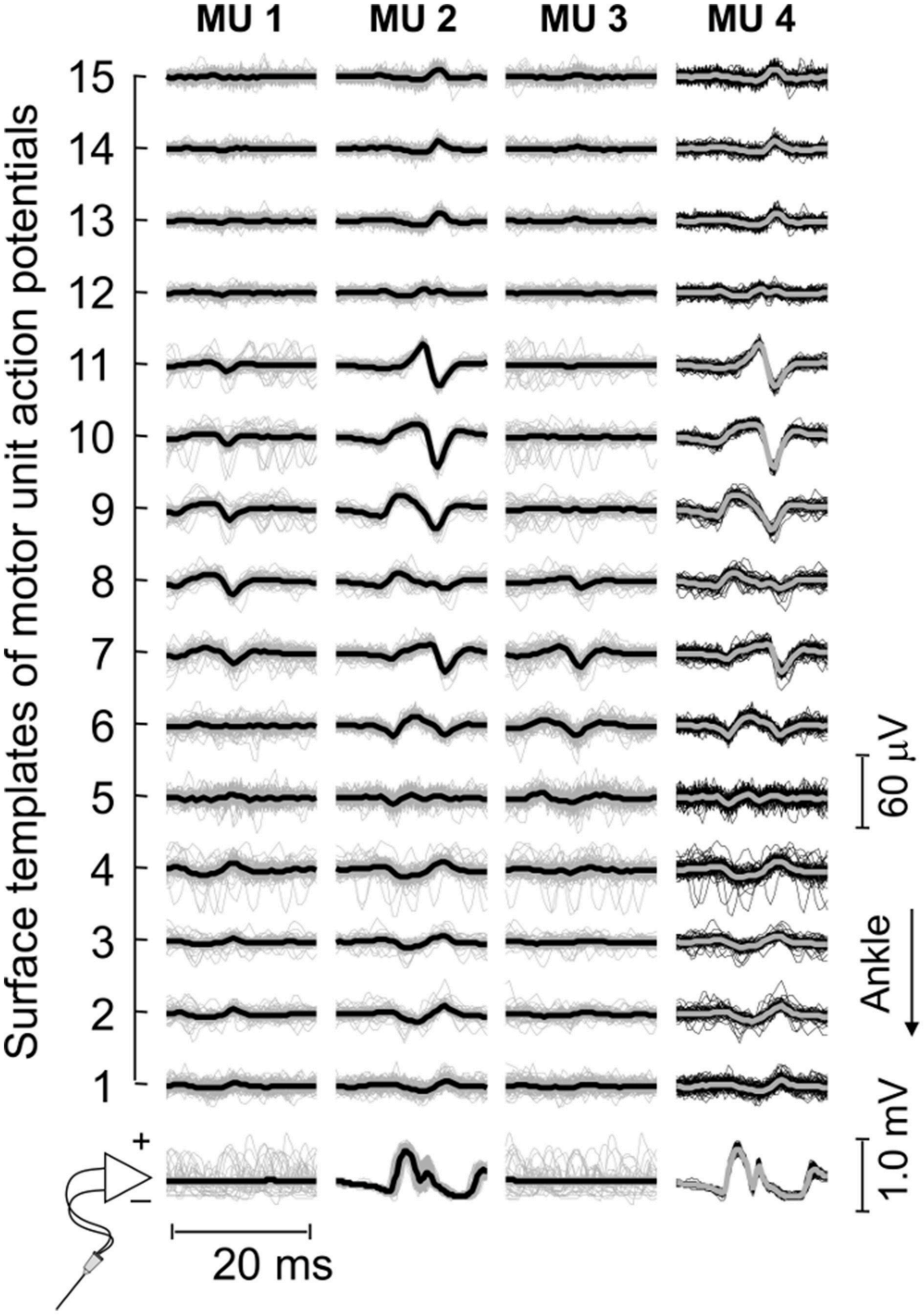
Motor unit action potentials in surface and intramuscular recordings. Short epochs (20 ms) of EMGs detected by wire and surface electrodes are shown. These epochs (thin traces) were obtained by triggering EMGs with the firing pattern of individual motor units, separately for each surface and intramuscular recordings. Thick traces were obtained by averaging the triggered EMGs. For this participant, three motor units were identified through decomposition of surface EMGs (cf. the first three columns from left to right). Note the similarity between waveforms obtained for the motor unit (MU 2) identified from decomposition of surface recordings and those obtained for the motor unit (MU 4) identified from decomposition of intramuscular EMGs.

First, EMGs were band-pass filtered with a fourth order Butterworth filter (cut-off frequencies; intramuscular: 500–3,000 Hz; surface: 20–400 Hz). Instants of motor unit firing were then automatically identified through decomposition of intramuscular EMGs (17). Often, a greater number of motor units could be observed in surface than intramuscular recordings (Figure 2D). To obtain the greatest number of motor units per subject, the firing pattern of additional motor units was identified through decomposition of surface EMGs with a validated procedure (18). It should be noted this decomposition algorithm does not rely on the shape but on the finite duration of motor unit action potentials. Even though its potential to reconstruct pulse trains decreases in highly underdetermined mixtures [i.e., when few electrodes are used (19)], here we assess the surface representation and not the firing pattern of single motor units. In virtue of the gastrocnemius pennate architecture, action potentials of different motor units appear in different locations and with different shapes on the skin (11, 20), making it possible to distinguish different motor units in the surface EMGs (e.g., Figure 3). Given the degree of gastrocnemius activity fluctuates during standing (11), the quality of decomposition results should be not assessed with conventional metrics as e.g., the coefficient of variation of the inter-spike intervals. For this reason, we used the pulse-to-noise ratio to assess decomposition accuracy (21); this metric does not depend on how regularly motor units discharged.

Decomposed EMGs were considered to assess side differences in the distribution of muscle units following stroke. Muscle units' distribution was estimated based on the surface amplitude of action potentials, using a slightly modified approach to that described in our previous study (11). Briefly, we first averaged EMGs over 40 ms epochs, centered on the firing instants of each motor unit. The root mean square (RMS) amplitude was then calculated for each of the 15 averaged EMGs, providing the surface distribution of RMS amplitude values for individual motor units. As shown previously (11), the distribution of RMS values scales with the distribution of fibers within individual, MG muscle units; the more distributed the muscle unit is, the more diffusely the RMS values distribute on the skin. After that, we computed Gaussian curves that numerically minimized the mean square error (MSE) with respect to the RMS values obtained for EMGs detected over MG superficial aponeurosis:

$$\begin{array}{l} MSE\left(\mu ,\sigma ,A\right)={\overset{15}{\underset{i=1}{\sum }}\left(RMS_{i}-\left[e^{-\frac{{\left(i-\mu \right)}^{2}}{2\sigma^{2}}}+A\right]\right)}^{2} \end{array}$$

where RMSi corresponds to the RMS amplitude obtained from the i-th channel, normalized with respect to the maximal RMS value across the array, whereas A was allowed to vary from 0 to 0.5 at 0.01 steps. The mean value (μ) of the Gaussian fitting spanned the RMS peak position ±2 cm and the theoretical standard deviation (σ), henceforth referred to as sigma, varied from 0.1 to 8 cm, both at 0.1 cm steps. *Sigma* values were then considered to assess how diffusely RMS values distributed on the skin [cf. Figure 3 in (11)]. Finally, *sigma* values were normalized with respect to the length of MG superficial aponeurosis (Figure 1B) to control for both side differences in muscle length (due e.g., to atrophy) and inter-individual differences. The normalized *sigma* values provide, therefore, a relative indication of how largely the amplitude of motor unit action potentials distributes along the MG physiological cross-sectional area [cf. Figure 1 in (22)].

**Figure 4:**
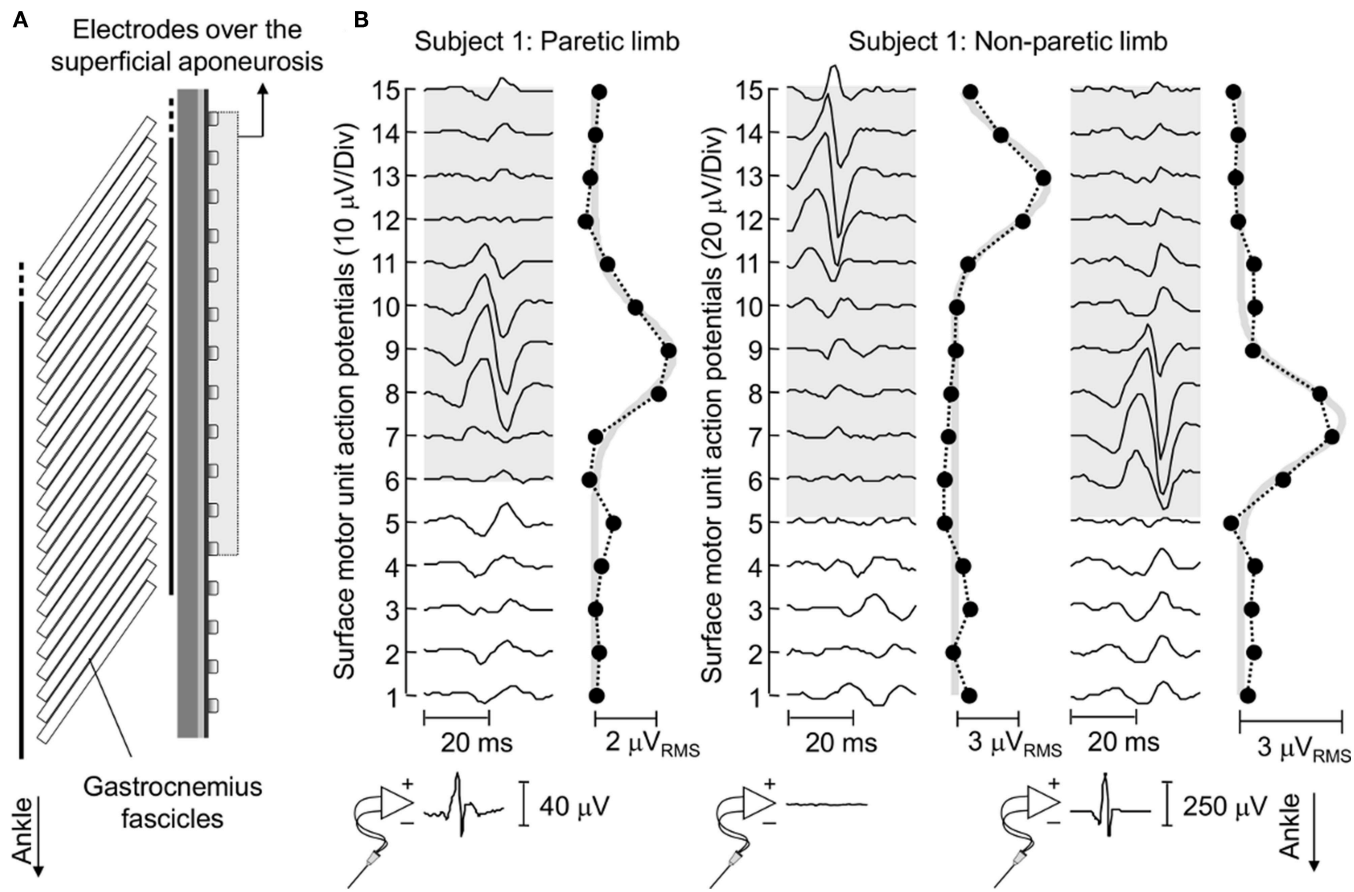
Surface representation of motor unit action potentials. **(A)** schematically illustrates the relative position between surface electrodes and MG fascicles for a representative subject. **(B)** shows the spike triggered average potentials of motor units identified for EMGs detected from the paretic and non-paretic MG muscle, both from the skin surface and intramuscularly. The absence of an action potential in the bottom trace of the central panel indicates this motor unit was identified through decomposition of surface EMGs. Circles denote the root mean square (RMS) amplitude of each surface EMG. Note the greatest RMS values appear in correspondence of EMGs conveying the biggest potentials. Thick gray traces correspond to Gaussian curves fitted to the distribution of RMS values (11). These curves were estimated by considering the RMS values of surface EMGs detected by electrodes over the superficial aponeurosis (shaded area).

The procedure considered above for the computation of *MSE* values is slightly different from that used in our previous study (11), where *A* was fixed at 0. This modified Gaussian curve allows for the compensation of baseline values different from 0, due both to noise and to the background activity not suppressed by averaging (Figures 3, 4). To assess potential differences in the quality of the Gaussian fitting between limbs, the coefficient of determination *R*^2^ was computed and then adjusted for the degree of freedom of the RMS variance (*dof*_*RMS*_ = 15 − 1 = 14) and of the estimated error variance:

$$\begin{array}{l} R_{adj}^{2}=1-\left(1-R^{2}\right)\frac{dof_{RMS}}{dof_{e}} \end{array}$$

### Muscle Architecture

Ultrasound images were acquired longitudinally from MG, with the US probe roughly centered where wire electrodes were inserted (7 MHz, 59 mm linear probe, Echoblaster 64, Telemed, Vilnius, Lithuania). Subcutaneous and MG thicknesses were quantified for the paretic and non-paretic limbs. The first was defined as the distance between the superficial aponeurosis and the skin-fat interface, whereas MG thickness was quantified as the distance between the superficial and deep aponeuroses.

### Statistics

Given data distribution was not Gaussian (Shapiro-Wilk test, *P* < 0.03 in all cases), the Mann-Whitney test was applied to quantify the significance of side differences in *sigma* values, in the adjusted coefficient of determination and pulse-to-noise ratio. Differences in the thickness of subcutaneous and MG tissues between limbs were quantified with the paired, Wilcoxon test. Skipped-Spearman correlation analysis (23) was used to assess whether there was a monotonic, positive relationship between the ratio values (paretic/non-paretic) for *sigma* and subcutaneous thickness.

## Results

### Gastrocnemius Motor Units Identified During Standing

Thirty-four motor units (13 in the paretic MG) were identified from EMGs collected from both limbs for the seven participants analyzed. The median number of motor units identified per subject in the paretic and non-paretic sides was, respectively, 2 (range: 1–3) and 3 (1–4). As shown in Figure 2, more units were decomposed from surface than intramuscular EMGs; close inspection of Figure 2B reveals that action potentials detected distally (from channel 3 to 5 at the first 50 ms) and proximally (from channel 9 to 11) belonged to different motor units, with the proximal though not distal potentials being detected by intramuscular electrodes as well (Figure 2D). When considering all participants, 16 and 20 motor units were decomposed from intramuscular and surface EMGs, respectively (two common units; Figure 3). Pulse-to-noise-ratio values were remarkably high for all units decomposed, varying from 25.3 to 36.5 dB (median value: 32.7 dB) for the non-paretic muscle and from 23.4 to 33.4 dB (30.4 dB) for the paretic muscle. No side differences in the pulse-to-noise ratio were observed (Mann-Whitney; *P* = 0.49).

Median values (interquartile intervals) for the adjusted coefficient of determination were 0.72 (0.60–0.89; *N* = 13 units) for the paretic and 0.81 (0.68–0.90; *N* = 21 units) for the non-paretic limb. No significant side differences were observed in the adjusted coefficient of determination (Mann-Whitney; *P* = 0.39), indicating the quality of Gaussian fitting in both sides was comparable.

### Side Differences in the Spatial Distribution of Muscle Units

Action potentials in the paretic and non-paretic MG were represented locally in the surface EMGs. As shown for a representative participant in Figure 4, action potentials with high amplitude were detected by few consecutive channels, centered at different skin regions. This local representation resulted in RMS values distributed narrowly on the skin, leading to Gaussian curves with relatively small *sigma* values in both non-paretic (0.79 and 0.88 cm) and paretic (1.05 cm) muscles. Due to a shorter aponeurosis in the paretic MG (cf. shaded area in Figure 4), relative side differences were emphasized by normalizing *sigma* with respect to the length of MG superficial aponeurosis (normalized *sigma*; paretic MG: 6.3%, non-paretic MG: 3.8 and 4.2%).

**Figure 5:**
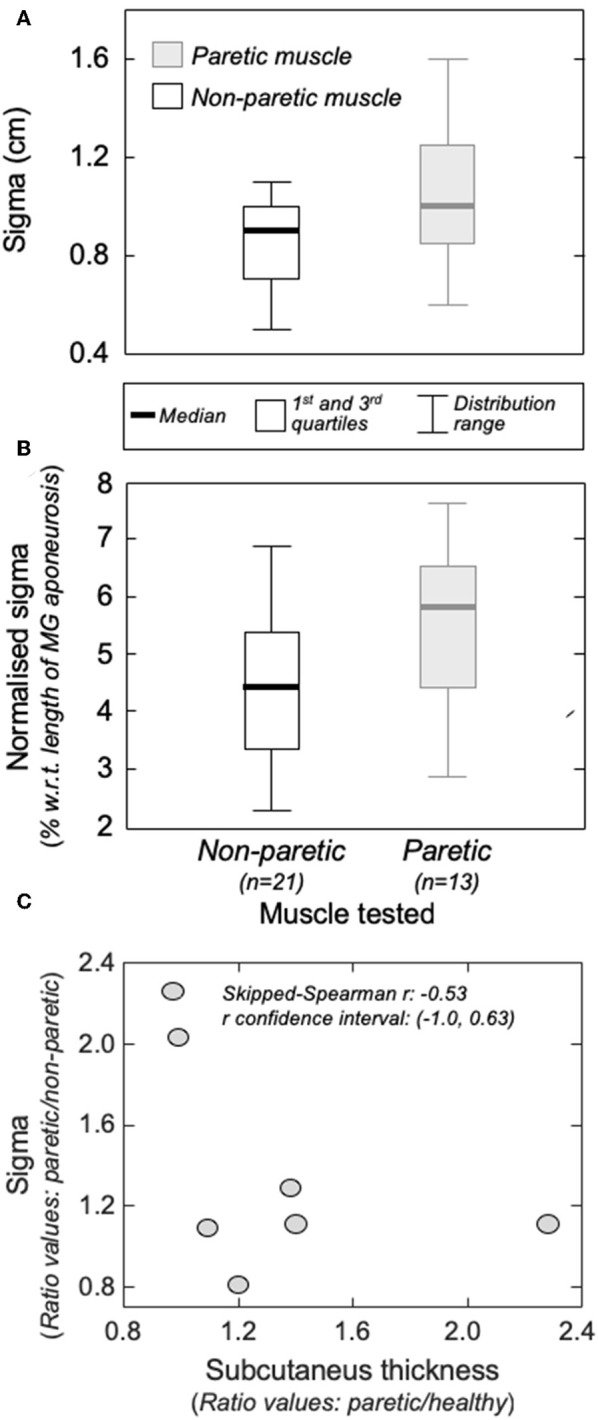
The amplitude distribution of surface, action potentials. **(A)** shows the standard deviation (*sigma*) of the Gaussian curves fitted to the RMS distribution of motor unit action potentials identified from the non-paretic (white boxes) and from the paretic (gray boxes) MG muscles. *Sigma* values normalized with respect to the length of the MG superficial aponeurosis (see Figure 1B) are shown in **(B)**. Thick horizontal lines denote the median values. Boxes and whiskers correspond, respectively, to the interquartile interval and the range values. Ratios between *sigma* values obtained from the paretic and non-paretic MG (ordinate) and ratios between subcutaneous thickness values computed for the paretic and non-paretic limb (abscissa) are shown in **(C)**. The skipped-Spearman correlation coefficient and its confidence interval (23) are shown within **(C)**.

Group analysis confirmed that action potentials of motor units in the paretic MG were represented over a significantly larger skin region than those from the non-paretic MG. The median *sigma* value in the paretic limb was significantly greater than that obtained for motor units in the non-paretic muscle (Figure 5A; Mann-Whitney; *P* = 0.037; *N* = 34 motor units). Side differences increased when normalizing *sigma* with respect to the length of MG superficial aponeurosis (Figure 1), measured separately for the paretic (range: 15–21 cm) and non-paretic limbs (16–22 cm). After normalization, the difference between median values in relation to the median *sigma* value in the paretic limb increased from ~11% (Figure 5A) to ~33% (Figure 5B; *P* = 0.014).

### Gastrocnemius and Subcutaneous Thickness

**Figure 6:**
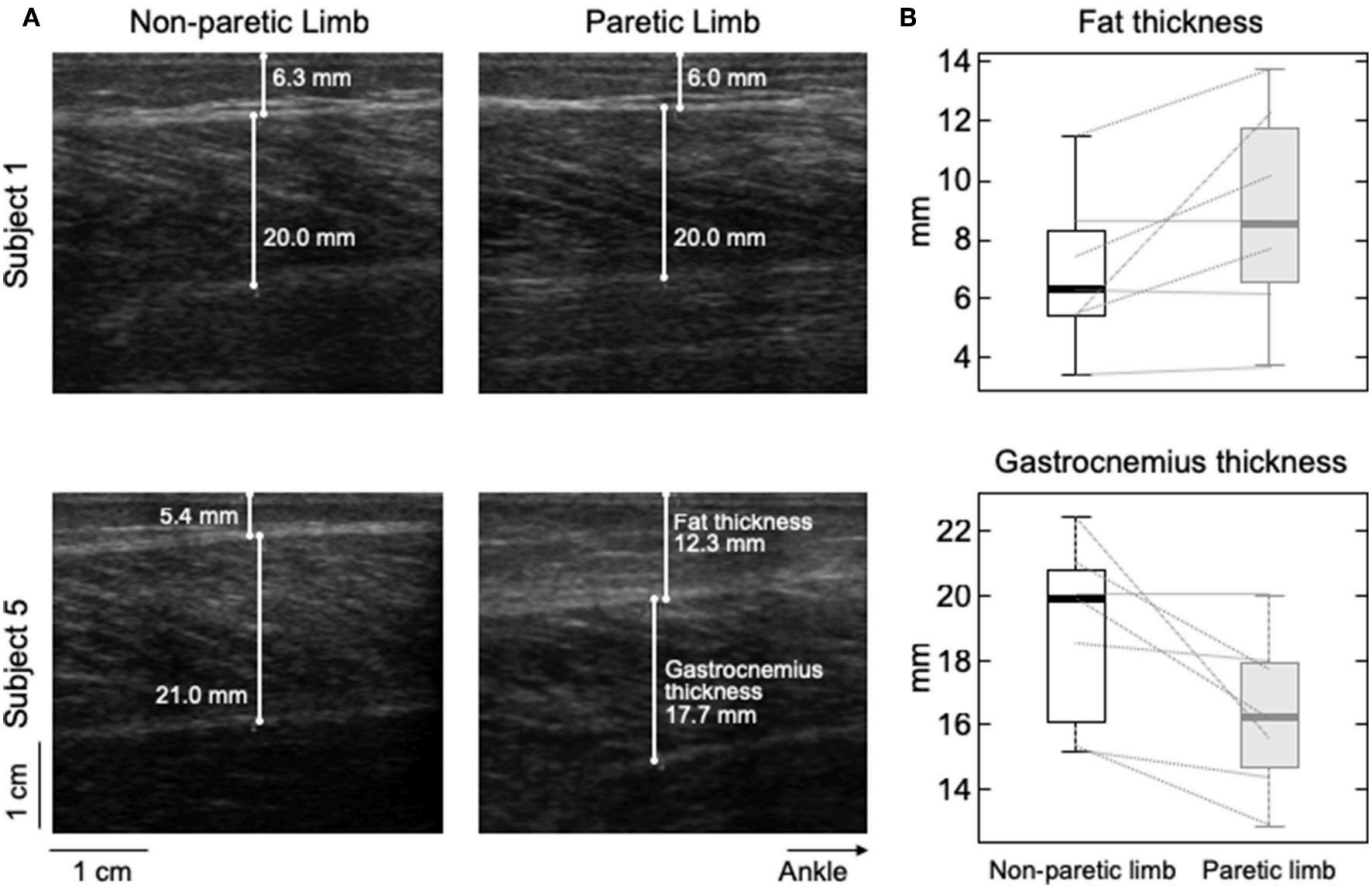
Differences in subcutaneous thickness between limbs. Ultrasound images recorded from the calf of two participants are shown in **(A)**. Images in the right and left were, respectively, taken from the paretic and non-paretic limb. The white lines superimposed on each image indicate the thickness of fat and MG tissues. **(B)** shows the distribution of subcutaneous (top) and gastrocnemius (bottom) thicknesses estimated for the seven participants. Thick horizontal lines denote the median values. Boxes and whiskers correspond, respectively, to the interquartile interval and the range values.

Different participants showed different degrees of structural muscle adaptations following stroke. For example, MG and subcutaneous thicknesses were similar in both limbs for subject 1 though not for participant 5, who showed thicker subcutaneous and muscle tissues for the paretic and non-paretic MG, respectively (Figure 6A). Notwithstanding these inter-individual differences, significantly thicker subcutaneous (Wilcoxon test; *P* = 0.046; *N* = 7 subjects) and thinner MG (*P* = 0.020, Figure 6B) tissues were observed in the paretic limb. No significant correlation was observed, however, for the paretic/non-paretic ratio values between *sigma* and subcutaneous thickness (Figure 5C; skipped-Spearman *r* = −0.53; confidence interval for *r* ranging from −1.00 to 0.63).

## Discussion

In this study, we combined intramuscular and surface EMGs to investigate structural changes in MG muscle units with stroke. Our results show action potentials of individual, postural muscle units were represented in relatively larger skin regions in the paretic than non-paretic MG (Figures 4, 5). Below we discuss the possible interpretations and potential implications of our findings.

### The Surface Representation of Motor Unit Action Potentials Changes With Stroke

Enlarged distribution of muscle units after stroke seems the most plausible explanation to our current findings. As evidenced by electrophysiological studies, structural changes within the neuromuscular system may be characterized by two distinct processes, occurring at different time periods after stroke. For example, a few weeks after stroke, Lukács (8) elicited smaller compound action potentials from hand muscles in the hemiparetic than non-paretic side. Similarly, the number of motor neurons estimated by Arasaki et al. (5) in the hypothenar muscles of the affected side started to decrease within the first 30 h following infarction. These findings suggest a reduction in the number of motor neurons in the acute phase post-stroke. A restoration process seems to commence though at chronic stages, whereby surviving motor neurons innervate the residual muscle fibers (6, 7), increasing the number of fibers per motor unit in the paretic side (14). The open question is whether reinnervated fibers distribute locally or diffusely within, and possibly beyond the confines of, the territory of restructured units. Based on results reported in Figures 4, 5, surface potentials of single MG units are represented in a significantly larger proximo-distal region in the affected than non-affected side.

The amplitude distribution of MG surface potentials is mainly affected by two factors: the distribution of fibers within the motor units' territory and the subcutaneous thickness. Because of the MG in-depth pinnation, the superficial extremity of different MG fibers is located beneath different, proximo-distal skin sites (e.g., Figure 4). Surface electrodes positioned closer to the distal extremity of active fibers detect therefore greater potentials. For this reason, the standard deviation (*sigma*) of Gaussian curves fitting the amplitude distribution of surface potentials was observed to scale with the number and location of fibers of MG muscle units [cf. Figure 4 in (11)]. Specifically, muscle units distributed locally were observed to provide surface potentials with high amplitude values distributed narrowly along MG (i.e., small *sigma* values). While the possibility of quantifying the perimeter of the territory of MG motor units with this technique could be questionable (24), it seems to provide a clear indication on the distribution of muscle units (25). Side difference in subcutaneous thickness is a potential, competing cause for obtaining greater *sigma* values in the paretic limb (Figures 4, 5). Because of the tissue filtering effect, the amplitude of surface potentials decreases with the distance between intracellular action potentials and the skin (26); i.e., the subcutaneous thickness. Although we observed thicker subcutaneous layer in the paretic limb (Figure 6), corroborating previous evidence on thigh muscles (27), it unlikely explains the side differences in sigma values between limbs (Figure 5). If this were the case, in opposition to results shown in Figure 5C, we would expect to observe greater sigma values for subjects with thicker subcutaneous tissue. It seems, therefore, the wider representation of motor unit action potentials in the paretic gastrocnemius is more likely due to redistribution of muscle units rather than subcutaneous thickness.

A note here is important on whether sources other than the redistribution of muscle units could have affected their representation in the surface EMGs. In non-paretic muscles, larger motor units are expected to convey a greater number of muscle fibers, spanning a presumably larger region of the muscle physiological cross-sectional area (9). Nevertheless, there is neurophysiological evidence of selective degeneration of the large (high threshold) motor units chronically after a stroke (28). Here, we could not control for side-differences in the size of motor units, even because we hypothesized the size, that is the innervation ratio, of motor units in the paretic limb had increased after stroke (6, 8). However, motor unit action potentials were often not observed in the paretic limb. Only after subjects were provided with CoP feedback and asked to voluntarily transfer their weight a few units could be appreciated in the surface EMGs (Figure 2). Visual inspection of surface EMGs detected from both limbs further revealed a smaller number of units in signals detected from the paretic than non-paretic limb.

Notwithstanding the small number of units decomposed from both limbs, two reasons suggest these units belonged likely exclusively to the gastrocnemius. First, the representation of motor units from soleus muscles is expectedly negligible in differential surface EMGs collected with inter-electrode distances as small as that used here [1 cm; (29)]. Second, false positives resulting from the decomposition algorithm should be <5% (21), considering the average pulse-to-noise ratio values obtained for both limbs. Therefore, it seems reasonable to state the motor units recruited in both limbs were the first recruitable or most excitable units in the gastrocnemius, suggesting like-with-like comparisons are assessed in results presented in Figure 5.

As discussed above, the enlarged representation of surface potentials suggests that MG muscle units span a relatively larger region in the paretic side. Advancing any mechanisms potentially accounting for such spatial enlargement with stroke would be currently speculative and is beyond the scope of the present study. It is, however, worth noting that, during the reinnervation process, the region occupied by single muscle units in the cat MG was reported to depend on how proximally the regenerating axon branches within the nerve (30); if branching occurs before the main nerve trunks, the regenerating axon may establish large territories. Despite the mechanisms accounting for the enlargement of muscle units, here we show the action potentials of single MG motor units are represented in larger proximo-distal regions in the affected (Figure 5B) than the non-affected side.

### Potential Implications of Enlarged, Muscle Units

Impaired control of standing has been reported for stroke survivors, as evidenced by large CoP displacements and asymmetric weight distribution (31, 32). While the etiology of stroke-induced, balance disorders is debatable (13), alterations of motor unit firing properties have been reported during standing. In stroke survivors exposed to postural disturbances, for instance, motor units show delayed activation (15), reduced synchronization within- and between-muscles (33) and prolonged inter-spike intervals (16). Our results seem to reveal a new marker of neuromuscular plasticity following stroke, the enlargement of MG muscle units. Considering MG contributes both to ankle plantar flexion and inversion (34, 35), enlarged muscle units may result in impaired regulation of force direction, possibly affecting the control of forward-backward and lateral body sways (36). Similarly, enlarged muscle units may result in a more uniform distribution of intramuscular pressure, compromising blood flow within MG (37). While further investigation is necessary to assess how changes in muscle unit distribution affect MG function, current results suggest muscle units may occupy a greater proportion of MG volume in the paretic limb of stroke survivors.

### Future Perspectives and Limitations

An open question arising from this study concerns the generalization of current findings to different populations of muscle units and to different muscles. With the possibility of assessing motor units from surface EMGs collected with grids of electrodes (18), the generalization of current results to a broader population of MG units could be tested in more demanding and controlled conditions (e.g., isometric contractions). We, nevertheless, specifically focused the analysis on motor units recruited during standing, which are presumably the smallest within MG (11). Moreover, even though the median number of units identified here was roughly half of that reported in previous studies on healthy subjects (11, 24), multiple surface EMGs collected along MG were all of markedly low amplitude. Whenever a motor unit fired during standing, its action potentials were clearly appreciated in the surface EMGs (cf. Figure 2). The relatively low number of motor units identified in the present study may be a consequence of the active loading of muscles other than MG during standing in stroke survivors. Results presented here seem therefore to be representative of MG motor units in stroke survivors, at least of those recruited during standing.

One limiting issue we did not address here is whether side differences in the surface representation of MG potentials exist in healthy subjects. Stroke has been shown, for example, to affect the firing rate of motor units even in the non-paretic limb, possibly because of differences in corticospinal excitability between limbs in stroke survivors (38). It should be noted, however, the metric we used here is not sensitive to side differences in the synaptic drive; the spatial representation of motor units in the surface EMG depends on the number and location of their action potentials and not on how frequently action potentials are discharged. Moreover, it should be emphasized that, irrespective of any potential (mal)adaptation that the non-paretic limb could suffer because of e.g., long-term excessive usage, properties such as peak torque, rate of force development, and voluntary activation (via twitch interpolation techniques) have been shown to differ from those observed in the paretic limb (39, 40). Our results, therefore, adds by showing one potential mechanism for the uneven state of the paretic related to the non-paretic limb.

## Data Availability

The raw data supporting the conclusions of this manuscript will be made available by the authors, without undue reservation, to any qualified researcher.

## Ethics Statement

This study was carried out in accordance with the recommendations of IDOR Institutional Ethic Committee with written informed consent from all subjects. All subjects gave written informed consent in accordance with the Declaration of Helsinki. The protocol was approved by the IDOR Institutional Ethic Committee (reference number 13611913.8.0000.5249).

## Author Contributions

TV, LO, and ER: conception and design of the experiments. TV, TL, LO, CH, and ER: collection, analysis, and interpretation of data. TV, TL, LO, GF, FT-M, and ER: drafting the article or revising it critically for important intellectual content.

### Conflict of Interest Statement

The authors declare that the research was conducted in the absence of any commercial or financial relationships that could be construed as a potential conflict of interest.
